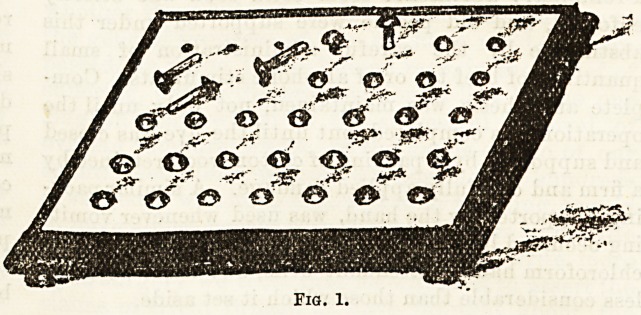# The Educational Treatment of Idiots, Imbeciles, and Feeble-Minded Children

**Published:** 1894-05-05

**Authors:** G. E. Shuttleworth

**Affiliations:** late Medical Superintendent, Royal Albert Asylum, Lancaster; formerly Assistant Medical Officer, Earlswood Asylum


### The Educational Treatment of Idiots, Imbeciles, and Feeble-Minded Children.

By G. E. Shuttleworth, B.A., M.D., &c., late Medical Superintendent, Royal Albert Asylum, Lancaster; formerly Assistant Medical Officer, Earlswood Asylum.

II.

In our last article (March 24th, 1894), we traced the history of the early efforts for the amelioration of the imbecile class in Europe. It would not be right, however, to omit to mention that almost simultaneously work was being done in the same direction in America. Early in 1846 the legislature of Massachusetts appointed a commission to inquire into the number and condition of idiots in the commonwealth, and whether anything could be done for their relief. Dr. S. G. Howe, the able superintendent of the Boston Blind School (immortalised in Dickens' "American Notes" as the successful instructor of the blind deaf-mute, Laura Bridgman), was chairman of the commission, and an exhaustive report was issued in 1848. This led to a grant by the legislature of 2,500 dollars for the establishment of an "experimental school for feeble-minded children, and thus the first American State Institution was opened in 1848. The idea "caught on," and, according to a recent report, there were in the United States at the end of 1892 nineteen public institutions maintained wholly or in part by State grants for educable feeble-minded, and in them, under care and training, an aggregate of 6,009 children. This is a striking contrast, not creditable to the old country, to the fact that in England and Wales what is analogous to State provision, that is to say, special institutions established or being established by the poor law, accommodate less than 1,600 educable imbeciles, whilst the five charitable institutions accommodate about an equal number. If expense be regarded as an objection to the extension of such accommodation in England, it may be urged that for some years past the comparatively poor countries of Norway and Denmark have contributed largely from State funds to special establishments for the instruction of the feeble-minded.

Passing now to a consideration of the principles upon which the education of imbeciles is conducted, we may quote from Séguin the fundamental axiom that "the education of the senses must precede the education of the mind." This principle, though universally accepted now, was by no means the pedagogic practice of fifty years ago. It is not too much to say, indeed, that general education has gained considerably from the results of experiments made on abnormal children. So-called learning by rote, after the manner of the parrot, was obviously inadequate to interest the imperfect intelligence of such. In many cases, moreover, all access to the central intelligence was barred by imperfections, sometimes organic, more often functional, of the external senses. Then again, with all there was more or less defect of the faculty of attention. To improve the latter by exercises attractive to child-nature, to promote ready obedience, and to bring the muscles into subjection to the will by simple drill; to quicken the senses by various ingenious devices, to cultivate clear perceptions, to train the hands to useful occupation, and finally to awaken the dormant moral faculties; these were some of the indications of the lines on which education must proceed. The problem was, moreover, often complicated by serious physical defects—paralytic symptoms, on the one hand, spasmodic movements on the other; and the occurrence of epilepsy in a considerable number of cases, would have discouraged any ordinary teacher from the irksome task. Séguin, however, had the advantage of a medical training, and he resolutely set to work to disengage and develop the mind of the idiot, which had hitherto been "as if hidden beneath the useless muscles and insensate nerves, components of his weak and inefficient body." Methodical exercises of the muscles, specially addressed to those which acted imperfectly, formed Séguin's starting-point, and drill of a simple character still forms in the "imbecile school," the stepping stone to education. Attention having been attracted, and imitation in certain directions secured, a series of what Séguin called "sensorial gymnastics" followed. Touch, sight, hearing, and even taste and smell were systematically tested, and where deficiencies were noted, efforts were made to counteract these. Ingenious exercises were devised to combat incapacities; the child with imperfect tactile power was coaxed to pick up pins and stick them into a pincushion, or place nails in the holes of a board perforated to receive them (see Fig. 1).

Fig. 1.

Sight, in many cases impeded by oscillating eyeballs (nystagmus) or incessant restlessness, was steadied by directing the gaze to sparkling objects such as mirrors and silvered balls, and the teacher was counselled to fix the pupil with his own eye, for, as Séguin quaintly puts it, the best method of "fixing the regard is the regard." Hearing is cultivated not only through the teacher's voice, but by means of music, the rhythm of which possesses a singular attraction for imbeciles. Tasting and smelling contrasting substances, similar in appearance (*e.g.*, salt and sugar, pepper and snuff), formed exercises for these senses. Contemporaneously, much attention was given to the cultivation of clear, articulate speech, deficient to a greater or less extent in the majority of idiots. It will be seen, therefore, that the system introduced by Séguin went entirely counter to the then prevalent practice of teaching merely through the memory, and it is interesting to note that the futility of such a method in the case of the feeble-minded is recognised by the poet Longfellow, who says ("Tales of a Wayside Inn"),

> As in an idiot's brain remembered words
> Hang empty 'mid the cobwebs of his dreams.

Throughout, the system propounded by Séguin is extremely practical; *things* are demonstrated, not merely *names* taught, in accordance with the Horatian maxim.

> Segnius irritant animos demissa per aurem
> Quam quæ sunt oculis subjecta fidelibus.

(*To be continued.*)

## Figures and Tables

**Fig. 1. f1:**